# Supplementary material for: Mechanisms governing the pioneering and redistribution capabilities of the non-classical pioneer PU.1
Source: Nat Commun. 2020 Jan 21;11:402. doi: 10.1038/s41467-019-13960-2 (PMC6972792; doi:10.1038/s41467-019-13960-2)
Supplement: Supplementary file 7 — Source data [file 41467_2019_13960_MOESM7_ESM.zip › Source_Data/FigureS4/FigureS4G_motifAnnotation/bound.pairs.PU1masked/knownResults.html]

/misc/data/analysis/project\_PU1/CTV1/motifs/basic/PU1.ntag15.filtered.12-50-bp.cluster.PU1masked - Homer Known Motif Enrichment Results


# Homer Known Motif Enrichment Results (/misc/data/analysis/project\_PU1/CTV1/motifs/basic/PU1.ntag15.filtered.12-50-bp.cluster.PU1masked)

Homer *de novo* Motif Results  
Gene Ontology Enrichment Results  
Known Motif Enrichment Results (txt file)  
Total Target Sequences = 10145, Total Background Sequences = 34248

|  |  |  |  |  |  |  |  |  |  |  |  |
| --- | --- | --- | --- | --- | --- | --- | --- | --- | --- | --- | --- |
| Rank | Motif | Name | P-value | log P-pvalue | q-value (Benjamini) | # Target Sequences with Motif | % of Targets Sequences with Motif | # Background Sequences with Motif | % of Background Sequences with Motif | Motif File | SVG |
| 1 | T G C A C T G A A G T C G T C A A C T G A C T G C G T A C G T A C T G A A G C T | EWS:FLI1-fusion(ETS)/SK\_N\_MC-EWS:FLI1-ChIP-Seq(SRA014231)/Homer | 1e-18 | -4.251e+01 | 0.0000 | 699.0 | 6.89% | 1575.4 | 4.60% | motif file (matrix) | svg |
| 2 | T A C G T C A G T G C A A G C T T G A C A G C T A G T C A C T G G A T C A C T G T C G A A C T G C T G A C T G A A T G C | ZBTB33(Zf)/GM12878-ZBTB33-ChIP-Seq(GSE32465)/Homer | 1e-13 | -3.146e+01 | 0.0000 | 58.0 | 0.57% | 41.3 | 0.12% | motif file (matrix) | svg |
| 3 | T C G A G A C T A G C T T G A C A G C T G T A C T C A G G A T C A T C G T G C A A C T G C T G A | GFX(?)/Promoter/Homer | 1e-12 | -2.893e+01 | 0.0000 | 43.0 | 0.42% | 23.4 | 0.07% | motif file (matrix) | svg |
| 4 | T C G A A G C T A C G T A C G T A G T C A G T C A C G T A T C G G A C T A T C G | EWS:ERG-fusion(ETS)/CADO\_ES1-EWS:ERG-ChIP-Seq(SRA014231)/Homer | 1e-9 | -2.111e+01 | 0.0000 | 831.0 | 8.19% | 2200.0 | 6.42% | motif file (matrix) | svg |
| 5 | C G T A T A G C T A G C T G C A A C T G C T A G C G T A C G T A T C A G G A C T | EHF(ETS)/LoVo-EHF-ChIP-Seq(GSE49402)/Homer | 1e-7 | -1.735e+01 | 0.0000 | 1295.0 | 12.76% | 3698.1 | 10.79% | motif file (matrix) | svg |
| 6 | T A G C G C T A T C G A C T G A A G T C A G T C C T G A A G T C C G T A C T A G | RUNX(Runt)/HPC7-Runx1-ChIP-Seq(GSE22178)/Homer | 1e-4 | -1.116e+01 | 0.0009 | 643.0 | 6.34% | 1794.9 | 5.24% | motif file (matrix) | svg |
| 7 | T G C A A G C T C T G A A T C G G A C T C T A G G T A C G A T C G T C A A G T C G T A C G A C T C T A G A T C G G C A T C A T G C A T G G A T C G T A C C T G A | CTCF(Zf)/CD4+-CTCF-ChIP-Seq(Barski\_et\_al.)/Homer | 1e-4 | -1.041e+01 | 0.0016 | 96.0 | 0.95% | 192.2 | 0.56% | motif file (matrix) | svg |
| 8 | T A C G T C A G A G C T A T G C C G T A A G T C T C A G A C G T A C T G T C G A | USF1(bHLH)/GM12878-Usf1-ChIP-Seq(GSE32465)/Homer | 1e-3 | -8.386e+00 | 0.0104 | 353.0 | 3.48% | 956.4 | 2.79% | motif file (matrix) | svg |
| 9 | A C T G G A T C G A C T A C T G A C G T C A T G A C T G A C G T A G C T C G A T | RUNX-AML(Runt)/CD4+-PolII-ChIP-Seq(Barski\_et\_al.)/Homer | 1e-3 | -8.317e+00 | 0.0104 | 666.0 | 6.56% | 1926.9 | 5.62% | motif file (matrix) | svg |
| 10 | C G T A C T G A C G T A C T A G T C G A C T A G A C T G C G T A C G T A T A C G A G C T A T C G | SpiB(ETS)/OCILY3-SPIB-ChIP-Seq(GSE56857)/Homer | 1e-3 | -7.949e+00 | 0.0128 | 19.0 | 0.19% | 20.7 | 0.06% | motif file (matrix) | svg |
| 11 | T C A G A G C T A T G C C G T A A G C T T C A G C A G T A C T G C T G A A G T C | MITF(bHLH)/MastCells-MITF-ChIP-Seq(GSE48085)/Homer | 1e-3 | -7.931e+00 | 0.0128 | 859.0 | 8.47% | 2546.8 | 7.43% | motif file (matrix) | svg |
| 12 | C T A G C A G T T G A C C G T A G A T C T C A G G A C T C A T G | BMAL1(bHLH)/Liver-Bmal1-ChIP-Seq(GSE39860)/Homer | 1e-3 | -7.131e+00 | 0.0243 | 1610.0 | 15.87% | 4996.8 | 14.59% | motif file (matrix) | svg |
| 13 | A T G C A T G C A T C G T A C G A G C T A G T C G C T A A G T C T C A G G A C T A C T G T C G A | E-box(bHLH)/Promoter/Homer | 1e-2 | -6.744e+00 | 0.0330 | 53.0 | 0.52% | 104.3 | 0.30% | motif file (matrix) | svg |
| 14 | G C T A C T G A T C G A A G T C A G T C C T G A A G T C G T C A C T G A T G C A | RUNX1(Runt)/Jurkat-RUNX1-ChIP-Seq(GSE29180)/Homer | 1e-2 | -5.584e+00 | 0.0976 | 995.0 | 9.81% | 3057.2 | 8.92% | motif file (matrix) | svg |
| 15 | C G A T T G C A G T A C C G T A A G T C C T A G G A C T C A T G | NPAS(bHLH)/Liver-NPAS-ChIP-Seq(GSE39860)/Homer | 1e-2 | -5.455e+00 | 0.1038 | 1370.0 | 13.50% | 4282.8 | 12.50% | motif file (matrix) | svg |
| 16 | C T A G C T A G A G T C T C A G A C T G A C G T A C G T C T G A | MYB(HTH)/ERMYB-Myb-ChIPSeq(GSE22095)/Homer | 1e-2 | -5.093e+00 | 0.1397 | 1529.0 | 15.07% | 4819.2 | 14.07% | motif file (matrix) | svg |
| 17 | C T G A T C A G A G T C C G T A A T C G A T G C C G A T A C T G A G T C G A C T A T C G A G T C | MyoD(bHLH)/Myotube-MyoD-ChIP-Seq(GSE21614)/Homer | 1e-2 | -4.972e+00 | 0.1483 | 559.0 | 5.51% | 1675.9 | 4.89% | motif file (matrix) | svg |
| 18 | T G A C G C T A T C G A T G C A A G T C A G T C C G T A A G T C C G T A C T G A G C T A G T A C | RUNX2(Runt)/PCa-RUNX2-ChIP-Seq(GSE33889)/Homer | 1e-2 | -4.910e+00 | 0.1491 | 742.0 | 7.31% | 2264.2 | 6.61% | motif file (matrix) | svg |
| 19 | G A C T C T A G C T A G A G T C T G C A A C T G A C G T A C G T C T A G T C A G | AMYB(HTH)/Testes-AMYB-ChIP-Seq(GSE44588)/Homer | 1e-2 | -4.787e+00 | 0.1597 | 1319.0 | 13.00% | 4145.6 | 12.10% | motif file (matrix) | svg |
